# The effect of posterior tethers on the biomechanics of proximal junctional kyphosis: The whole human finite element model analysis

**DOI:** 10.1038/s41598-020-59179-w

**Published:** 2020-02-26

**Authors:** Mitsuru Yagi, Yuko Nakahira, Kota Watanabe, Masaya Nakamura, Morio Matsumoto, Masami Iwamoto

**Affiliations:** 1 Department of Orthopedic Surgery, Keio University School of Medicine, Shinjuku-ku, Tokyo, Japan; 2 Department of Orthopedic Surgery, National Hospital Organization Murayama Medical Center, Musashimurayama city, Tokyo, Japan; 3 Toyota Central R&D Labs Inc., Nagakute city, Aichi, Japan; 4 Toyota Central R&D Labs Inc., Tokyo, Japan

**Keywords:** Skeletal muscle, Skeleton

## Abstract

Little is known about the effects of posterior tethers on the development of proximal junctional kyphosis (PJK). We evaluated the ability of posterior tethers to the proximal motion segment stiffness in long instrumented spinal instrumentation and fusion using a whole body human FE model. A series of finite element (FE) analysis of long segmental spinal fusion (SF) from the upper thoracic vertebra (T1) or lower thoracic vertebra (T9) to the sacrum with pedicle screws and rods were performed using an entire human body FE model (includes 234,910 elements), and compressive stresses (CS) on the anterior column, and tensile stresses (TS) on the posterior ligamentous complex (PLC) in the upper-instrumented vertebra (UIV) and the vertebra adjacent to the UIV (UIV + 1) were evaluated with posterior tethers or without posterior tethers. The models were tested at three T1 tilts (0, 20, 40 deg.), with 20% muscle contraction. Deformable material models were assigned to all body parts. Muscle-tendon complexes were modeled by truss elements with a Hill-type muscle material model. The CS of anterior column decreased with increasing T1 slope with tethers in both models, while the CS remained relatively large in T9 model compared with T1 model (T1 UIV; 0.96 to 1.56 MPa, T9 UIV; 4.79 to 5.61 MPa). The TS of the supraspinous ligament was markedly reduced in both T1 and T9 models with posterior tethers (11–35%). High vertebral CS on UIV and UIV + 1 were seen in the T9 UIV model, and the TS on the PLC were increased in both UIV models. Posterior tethers may decrease PJK development after SF with a proximal thoracic UIV, while both posterior tethers and vertebral augmentation may be necessary to reduce PJK development with a lower thoracic UIV.

## Introduction

Proximal junctional kyphosis (PJK) is a common but devastating complication after corrective spine surgery for spinal deformity, especially in patients with pre-existing low bone mineral density (BMD)^1–7^. The incidence of PJK after corrective spine surgery for adult spinal deformity (ASD) is 36–50% in most recent reports^1–7^. In these studies, 10–41% of the patients required revision surgery due to intolerable pain, difficulty of forward gazing, or paralysis^2,4–6^. Several studies have examined the causes and risk factors for this postoperative complication^1–7^. A large preoperative sagittal malalignment, large pelvic tilt, and large surgical restoration of lumbar lordosis are spinal-alignment-related risk factors for PJK development, while destruction of the soft tissue and low BMD are surgery- and patient-related risk factors, respectively^2,4,7–9^. Several prophylactic treatment strategies have been proposed to prevent PJK, including teriparatide administration and cement augmentation of adjacent vertebra^10–12^. However, since PJK appears to be a multi-factorial phenomenon, more research is needed, especially on the effects of spinal alignment and level of fusion. Although previous studies described the biomechanical aspects of PJK, little is known about the effects of instrumentation and sagittal spinal alignment on PJK development. Finite element (FE) models have been widely accepted and used to simulate the effect of fusion on the biomechanics of the human spine and limb joints^13–15^. Several studies described the effects of posterior fusion and destruction of the soft tissue on the adjacent segment^2,4,6–9^. Cahill *et al*. described the effect of instrumented fusion on the adjacent segment and concluded that pressure in the nucleus pulposus and angular displacement increased when the intraspinous ligament (ISL) and supraspinous ligament (SSL) complex were removed immediately above the instrumented levels in an adolescent model^15^. Very recently, Buell *et al*. described the junctional posterior tethers to reduce the occurrence of PJK in their pilot study^16^. Buell *et al*. also described the favorable biomechanical aspect of posterior tethers to prevent PJK.

However, all of the previous studies used regional spine FE models for analysis, and none demonstrated the effect of long instrumented spinal fusion on adjacent level pathologies using a whole-body simulation^17^. Therefore, to investigate the prevention strategy of the prevention of PJK using a whole-body simulation in a various spinal fusion level and sagittal spinal alignment is indispensable.

Toyota Central R&D Labs., Inc. and Toyota Motor Corporation jointly developed a whole human body FE model named THUMS (Total HUman Model for Safety) to predict kinematics and injuries of occupants or pedestrians during various crash situations seen in traffic accidents and reported on the applicability of the model^18–33^. A human body FE model used in this study was developed based on the THUMS Version 3^34^ by incorporating 282 individual muscle models of the whole body into the THUMS to investigate effect of muscle activity on kinematics and injuries of the whole body^35–37^. In this model, 3D geometry of each muscle is reproduced based on Visible Human Project data (National Institutes of Health, USA) and the stiffness of muscles could be changed according to muscle activation levels. In addition, multiple contacts are defined between muscles and between muscles and bones to reproduce natural and smooth movements of human body. Therefore, postures of this human body FE model could be easily changed. The human body FE model has been applied in various studies on impact biomechanics and medical studies^36–39^.

Overcoming PJK is an urgent goal, for which it is important to elucidate the pathomechanics involved. Therefore, the purpose of this study was to use the human body FE model to evaluate the biomechanical effects of posterior tethers for the level of upper-instrumented vertebra (UIV), the vertebra adjacent to the UIV (UIV + 1) under conditions including the various spinal alignment with muscle activity.

## Materials and Methods

The authors declare no conflicts of interest associated with this manuscript.

### FE model

An FE model of instrumented spinal fusion from the upper thoracic vertebra (T1) or lower thoracic vertebra (T9) to the sacrum with segmental bilateral pedicle screws and rods was created on the abovementioned human body FE model^35–37^ (includes 234,910 elements, Supplemental Figs. 1–3), to evaluate compressive stresses on the anterior column, and tensile stresses on the posterior ligamentous complex (PLC) within and adjacent to the UIV. This model is similar in size to AM50 (the 50-percentile adult male), with a height of 175 cm and a weight of 77 kg. The human body FE model can be available on a general-purpose FE program LS-DYNA (Livermore Software Technology, USA)^40^. Posterior tethers were add-on between to the UIV and UIV + 1 lamina (Supplemental Fig. 4).

In the human body FE model, the cancellous bones of the vertebral body are modeled using solid elements (hexahedron or tetrahedron) while the cortical bones are modeled using shell elements (quadrangle or triangle). The material properties of cancellous and cortical bones are modeled using an elastic-plastic material model to predict bone fracture risks. The ligaments are modeled using shell elements (quadrangle or triangle) or beam elements (line). The thicknesses of the ligaments are set for the shell elements and the cross-sectional areas are set for the beam elements. The material properties of ligaments are modeled using a nonlinear elastic material model. The discs are modeled using solid elements (hexahedron) in which the annulus is modeled using a rubber-like material model while the nucleus pulposus is modeled using a linear elastic material model to simulate human-like spinal motions^26^. Each muscle is represented as a hybrid model by combination of solid elements with passive muscle properties and bar elements with active muscle properties to simulate both human body motions and impact energy absorption. The passive muscle properties are modeled using a rubber-like material model while the active muscles properties are modeled using a Hill-type muscle model^35^. In this study, we used the human body FE model to predict injury risks of the vertebral bodies and spinal ligaments. The risks of bone fractures were evaluated using the maximum compressive stress distribution on the vertebral bodies while the risks of ligament ruptures were evaluated using the maximum tensile stress of the spinal ligaments.

### Calculation condition using UIV models

#### UIV models

**Figure 1:**
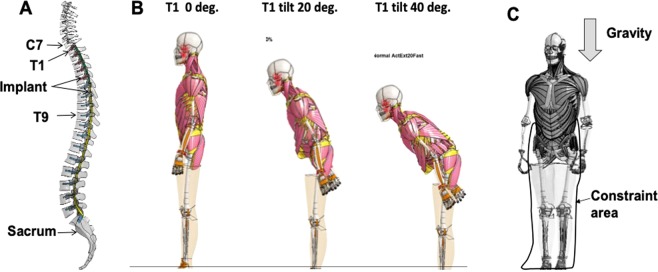
A human body FE model with solid muscle models. (**A**) Implant FE model incorporated into T1~Sacrum in case of T1 UIV. (**B**) Three different sagittal T1 tilts used in this study (0, 20, and 40 degrees). (**C**) Initial conditions of the calculation. The degree of freedom from the pelvis to the foot of the human body FE model in the standing posture was constrained, and gravity was loaded for 1 second.

We created two different UIV models: in one, the vertebrae below T1 were fused (T1 UIV), and in the other, the vertebrae below T9 were fused (T9 UIV). The models had instrumented spinal fusion from either T1 or T9 to the sacrum. Implant FE models were created to be incorporated into these fused ranges as shown in Fig. 1A. Because the implant usually used for spinal fixation is very stiff (cobalt chrome alloy: ASTM1357), a rigid material was applied to reduce the computation time.

To simulate a spinal deformity patient, especially one with a sagittal spinal deformity, an anteflexion calculation was performed using the human body FE model in a standing posture (body size of the human body FE model corresponds to that of an adult mid-size male; the mechanical properties of bone and muscles etc. correspond to those of a 40–49-year-old), and the first thoracic vertebra (T1) was tilted forward. The mean T1 slope of anteflexion patients is around 20 degrees from clinical observation^41,42^ whereas the T1 slope of around 40 degrees tends to have high fracture risk of adjacent spines^3–5,7^. Thus, we created three models with the T1 sagittal tilts of 0, 20, and 40 degrees to investigate effect of the T1 slope on the developing of PJK. We first extracted the node coordinates when the T1 sagittal tilts were 20 and 40 degrees. The human body FE model with three sagittal spinal alignments was then created by replacing the original coordinates by the extracted node coordinates. Figure 1B shows the two created models with T1 sagittal tilt angles of 20 and 40 degrees, and also shows a model with a T1 sagittal tilt angle of 0 degrees which is a normal alignment, as a control case for comparison. The model with the T1 sagittal tilt of 0 degrees had 33, 34, and 48 degrees in the cervical lordosis (CL), the thoracic kyphosis (TK), and the lumber lordosis (LL), respectively whereas the model with the T1 sagittal tilt of 20 degrees had 58, 41, and 30 degrees in CL, TK, and LL. In addition, the model with the T1 sagittal tilt of 40 degrees had 74, 28, and 6 degrees, respectively. A parameter study was conducted using these three models.

To simulate the body conditions of patients after spinal fusion, elements of the ligaments and the muscles within the fused range dissected during the surgery were deleted from all the three models. The splenius cervicis muscle, semi-spinalis cervicis muscle, semispinalis capitis muscle, longissimus cervicis muscle, longissimus capitis muscle, and the inter-transversalis ligament (ITL) below the seventh cervical vertebra (C7) were deleted in the T1-fused model, and the ITL below the eighth thoracic vertebra (T8) was deleted in the T9-fused model (see Supplemental Fig. 1).

#### Muscle activity

To investigate effects of muscle activity on PJK, it is necessary to input activation level of each muscle. In this study, we set a condition with muscle activity. While PJK could be developed in several postures, PJK could be developed during standing. In the case with muscle activity, an activation level was inputted only to the extensor muscles, to suppress the pre-flexion caused during relaxation. The maximum value of the activation was 20%, which is reported as the mean value of muscle activation levels in the trunk from electromyogram measurement data in the seated state^36^.

#### Calculation conditions

Two UIV levels (T1 to sacrum, and T9 to sacrum) were simulated at three different T1 tilts (0, 20, and 40 degrees) with muscle activity with or without posterior tethers. Therefore, 12 models were used for parametric simulations in this study. Additionally, no instrumentation model was used as the control model.

Figure 1C shows the initial conditions for the calculation. The degree of freedom from the pelvis to the foot of the human body FE model in the standing position was constrained, and the gravitational acceleration was applied to each element of the upper body except the constrained pelvis and lower limbs for 1 second. To reduce the computational time, the lower limbs and upper limbs were omitted. The skin of the trunk was modeled using solid elements with high stiffness in the original human body FE model to simulate skin characteristics with high deformation rates for impact simulations. However, such a model is not used to reproduce the skin characteristics when the deformation speed is slow, as in daily activity. Therefore, in this study, as shown in Fig. 1C, we used a model in which the skin of the trunk was deleted. As a result, the number of elements of the human body FE model used in this report was 234,910. The material properties of the posterior elastic tether were obtained based on the surgical ultrahigh molecule weight polyethylene tape from previous report^43^ (Young’s module: 3.5 GPa, Poisson’ ratio: 0.4, thickness: 0.5 mm, width: 7 mm).

## Hypothesis

Posterior tethers may reduce the tensile stress of PLC and compressive stress of the anterior column of UIV + 1 vertebra after instrumented spinal fusion.

## Results

### Spinal stress distribution in control and spinal fusion model with or without posterior tethers

**Figure 2:**
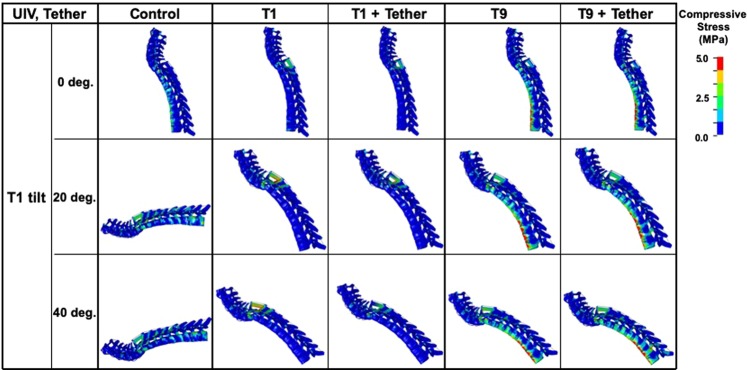
Stress distributions on T1-T9 vertebral column. Compressive stress distributions of the spinal column from C1 to T9 during maximal anterior bending. The compressive stresses on UIV and UIV + 1 were great in the T9 UIV and small in the T1 UIV model. High compressive stresses were observed on the vertebrae around the UIV in both the T1 and T9 UIV models. In particular, in the T9 UIV model, high compressive stress of about 5 MPa or more occurred on the anterior surface of the vertebral body.

Figure 2 shows compressive stress distributions of the spinal column of the Atlas (C1) to T9 during maximal anterior bending with or without posterior tethers. The compressive stresses on the UIV and UIV + 1 were high in the T9 UIV model and low in the T1 UIV model. High stress was observed on the vertebrae around the UIV in both the T1 and T9 UIV models. In particular, in the T9 UIV model, a high compressive stress level of about 5 mega-pascals (MPa) or more was present on the anterior surface of the vertebral body.

In T9 model with posterior tethers, the compressive stress of anterior column remained relatively high around the UIV (Fig. 2).

### Maximum compressive stress on the anterior column of vertebral body

**Figure 3:**
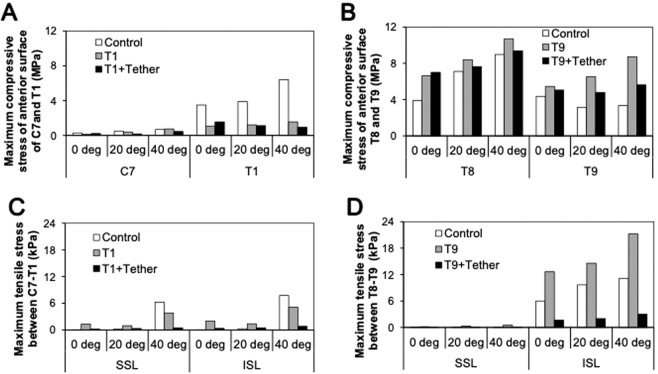
Maximum compressive stresses on the anterior column of the UIV and UIV + 1 vertebrae and tensile stress between at UIV and UIV + 1 at various T1 tilts on control, T1 UIV, and T9 UIV model with or without posterior tethers. (**A**) Maximum compressive stresses on the anterior column of C7 and T1 in the T1 UIV model with or without posterior tethers. (**B**) Maximum compressive stresses on the anterior column of T8 and T9 in the T9 UIV model with or without posterior tethers. (**C**) Maximum tensile stress of SSL and ISL between UIV and UIV + 1 at various T1 tilts on control, T1 UIV, and T9 UIV model with or without posterior tethers. (**D**) Maximum tensile stresses on SSL and ISL between UIV and UIV + 1 at various T1 tilts on control and T1 UIV model with or without posterior tethers.

Tables 1, 2 and Fig. 3 compare the maximum compressive stresses at the anterior columns of the UIV and UIV + 1 vertebrae between with and without the fusion under different T1 sagittal tilt angles, respectively. In the T9 UIV model, the largest compressive stress on UIV + 1 occurred when the T1 tilt angle was 40 degrees (Fig. 3B and Table 2). Comparison of the T1 and T9 UIV models showed that the compressive stress on UIV + 1 was approximately 15–50 times higher in the T9 UIV model than the T1 UIV model. The same trend was seen for the UIV in the T1 versus T9 UIV model, but the difference was about 3–6 fold (Tables 1 and 2).

**Table 1:** Maximum compressive stresses on the anterior column of the UIV and UIV + 1 vertebrae at various T1 tilts on T1 UIV model with or without posterior tethers.

Level	C7 (UIV + 1) [MPa]			T1 (UIV) [MPa]		
T1 tilt	Control	T1 UIV	T1 + Tether	Control	T1 UIV	T1 + Tether
0 deg.	0.29	0.13	0.22	3.50	1.08	1.56
20 deg.	0.52	0.40	0.19	3.89	1.21	1.13
40 deg.	0.68	0.75	0.45	6.41	1.57	0.96

**Table 2:** Maximum compressive stresses on the anterior column of the UIV and UIV + 1 vertebrae at various T1 tilts on T9 UIV model with or without posterior tethers.

Level	T8 (UIV + 1) [MPa]			T9 (UIV) [MPa]		
T1 tilt	Control	T9 UIV	T9 + Tether	Control	T9 UIV	T9 + Tether
0 deg.	3.88	6.62	7.01	4.35	5.46	5.05
20 deg.	7.12	8.40	7.63	3.15	6.52	4.79
40 deg.	8.98	10.71	9.39	3.35	8.73	5.61

In these cases, posterior tethers decreased the maximum compressive stresses on the anterior column of vertebral body. In the T1 UIV model, the compressive stresses of the anterior column decreased to 46–60% in UIV + 1 vertebra when the T1 tilt angle was greater than 20 degrees. Similarly, in the T9 UIV model, the compressive stresses of the anterior column decreased to 88–91% when the T1 tilt angle was greater than 20 degrees. Comparison of the T1 and T9 UIV models with posterior tethers showed that the compressive stress on the anterior column of UIV + 1 for the T9 UIV model was 20–40 times higher than that of the T1 UIV model.

### Maximum tensile stresses of PLC

When the spine sustains forward bending, the PLC (supraspinous ligament: SSL and intraspinous ligament: ISL) attached to the spinous process of the vertebral body is stretched (Supplemental Fig. 3). The tensile force divided by the cross-sectional area of each ligament was calculated as tensile stress of the PLC. Tables 3 and 4 in addition to Fig. 3C,D show the maximum tensile stresses on the SSL and ISL under the maximum forward bending. Compared with the control model, the tensile stresses were higher in both the T1 and T9 UIV models except for 40 degrees of the T1 UIV model. Posterior tethers significantly decreased the tensile stresses of SSL to 13–32% in T1 UIV model and 11-18% in T9 UIV model. Same trend was seen in the tensile stress of ISL (16–35% in T1 UIV and 13–14% in T9 UIV model).

**Table 3:** Maximum tensile stresses of SSL and ISL at various T1 tilts on T1 UIV model with or without posterior tethers.

Level	SSL [kPa]			ISL [kPa]		
T1 tilt	Control	T1 UIV	T1 + Tether	Control	T1 UIV	T1 + Tether
0 deg.	0.01	1.32	0.19	0.02	1.99	0.34
20 deg.	0.16	0.97	0.31	0.27	1.39	0.49
40 deg.	6.25	3.83	0.49	7.75	5.10	0.81

**Table 4:** Maximum tensile stresses of SSL and ISL at various T1 tilts on T9 UIV model with or without posterior tethers.

Level	SSL [kPa]			ISL [kPa]		
T1 tilt	Control	T9 UIV	T9 + Tether	Control	T9 UIV	T9 + Tether
0 deg.	0.06	0.16	0.03	5.99	12.64	1.65
20 deg.	0.06	0.30	0.04	9.67	14.59	2.00
40 deg.	0.01	0.51	0.05	11.16	21.20	2.99

## Discussion

PJK is multifactorial phenomenon with various reported risk factors, including both surgery- and patient-related factors^1–7^. Among the surgical factors, the sagittal spinal alignment and level of the UIV are well recognized risk factors for PJK development^2,4,7–9^. However, even though the odds ratios for these risk factors have been reported, the biomechanical aspects of the UIV level and the spinal alignment have not been fully understood. To determine the optimal spinal fusion surgery for a spinal deformity, it could be beneficial to apply a human body FE model including muscles, ligaments, and intervertebral discs, those could predict fractures. In the present study, we predicted the effects of posterior tethers on the adjacent segment of long spinal instrumentation and fusion with various UIV level and spinal alignment using an abovementioned human body FE model. We performed an FE analysis on long segmental spinal fusion using the whole human body model with instrumented fusion from either T1 or T9 to the sacrum, and assessed the effects of spinal fusion, UIV level, sagittal spinal alignment, and posterior tethers on the proximal junction.

### Stress distributions on spinal column

The compressive stresses were significantly concentrated on the anterior column at both UIV and UIV + 1 in all simulations. Comparisons of the stresses on UIV and UIV + 1 between the T1 and T9 UIV models showed higher spinal column stresses in the T9 UIV model and lower stresses in the T1 UIV model. The compressive stresses were increased with an increase in T1 sagittal tilt in both T1 and T9 UIV models as shown in Tables 1 and 2, suggesting that vertebral body loading is reduced by the restoration of overall spinal alignment.

High stresses were generally observed on the vertebrae around the UIV and in the PLC in both the T1 and T9 UIV models. It is likely that when the vertebral body is inclined forward due to sagittal spinal malalignment and the distance of the front face between the adjacent vertebral bodies is reduced, the adjacent vertebral bodies receive the compressive load. When the distance between the spinous processes on the posterior column of the vertebral body between UIV and UIV + 1 increased, a force occurred to stretch the ligaments (SSL and ISL) attached to the spinous processes (see Supplemental Fig. 3). In particular, in the T9 UIV model, a large stress of about 5 MPa or more occurred on the anterior column of the vertebral body. The vertebral fracture risk is reported to increase when the vertebral compressive stress is about 6 MPa or more in 40–59-year-old individuals^44^. PJK is common in elderly patients with pre-existing low BMD, and the models used in this study clearly showed that having the UIV at a lower thoracic vertebra is a significant risk factor for PJK development in these patients^5^.

### Maximum compressive stress on the anterior column of the vertebral body

As shown in Table 2, In the T9 UIV model, the largest compressive stress on UIV + 1 occurred when the T1 tilt angle was 40 degrees. One possible reason for the increased compressive stress in this situation is increase in compressive stress at UIV. The reason why compressive stress at the T1 tile angle of 40 degrees in both UIV models was increased compared to non-fixation control is probably because the angle between the gravity direction and the horizontal plane of the vertebral bodies in both UIV models became acute as the T1 sagittal tilt angle increased so that the relative stress applied to the UIV + 1 was increased because the UIV was fixed.

Comparison of the T1 and T9 UIV models showed that the compressive stress on UIV + 1 was approximately 15–50 times higher in the T9 UIV model than the T1 UIV model. These simulation results are consistent with the clinical observations that both UIV + 1 and UIV fractures occur when fusion to T9 is performed for adult spinal deformity patients. In addition, the compressive stress in the T1 UIV model was lower than that in the T9 UIV model. In the T1 UIV model, C7 (UIV + 1) only supports the head and there is no need to support the arm or the trunk in contrast to T9 fusion. Therefore, the compressive stress on the vertebral body is small. This finding is also consistent with the clinical observations that UIV and UIV + 1 fractures rarely occur when T1 is selected for the UIV.

### Maximum tensile stresses of PLC

As the degree of kyphosis decreases, the distance between the spinous processes of the upper and lower vertebral bodies becomes shorter, and SSL and ISL also become shorter proportionally; therefore, tensile stresses in the ligaments should decrease. Compared to the control models, the tensile stresses in the PLC were higher in the UIV models. This was probably because the fused vertebral body prevented from tilting forward, but the UIV + 1 could tilt forward, thereby increasing the difference in the anterior tilt angle between these two vertebral bodies.

The tensile stresses on the SSL were higher in the T1 UIV model than in the T9 UIV model, while the stresses on the ISL were higher in the T9 UIV model than in the T1 UIV model. Clinically, rupture of the SSL and ISL often occurs in T1 fixation. Compared with previous clinical studies on PJK, our results were consistent for SSL but not for ISL. It will be necessary to review the thickness, initial length, and material properties of the ligament to improve the prediction accuracy for injury to the ISL.

Simulation results with gravitational load demonstrate that the upper part of the body bends forward from the pelvis when the vertebral bodies are not fused while only the vertebrae from the head to a vertebra above the fused range bend forward when the vertebral bodies are fused. The maximal anterior bending caused significant stresses on the anterior column of UIV and UIV + 1 and on the ISL and SSL. Compared with the previous clinical studies on PJK, the regions where the greatest stresses occurred in the model used in this study coincided with the reported sites of fracture occurrence and ligament injury.

### Posterior tethers

Posterior tethers successfully decreased the compressive stress of the anterior column of UIV and UIV + 1 in both T1 and T9 UIV models (see Tables 1 and 2) in when T1 tilt was 20 and 40 degrees, however, the compressive stress was slightly increased when T1 tilt was 0 degree. We speculate that this is because the posterior tethers force functioned to reduce each interbody distance and increased the compressive stress especially the T1 tilt was 0 degrees. In this situation, the position of head was more posterior compared with the T1 tilt 20 and 40 degrees and therefore the compression stress of anterior column in the T1 tilt of 0 degree tended slightly large in anterior column of UIV and UIV + 1 vertebra compared with other models.

The posterior tethers significantly decreased the tensile stress of ISL/SSL in both UIV models. This finding was consisted with the previous preliminary report of posterior tethers for the incidence of PJK following ASD surgery. Buell *et al*. described that junctional tethers significantly reduced the occurrence of PJK from 45.3% to 34.4% following ASD surgery with minimum 3 months post-operative follow-up^17^.

In this study, high vertebral compressive stresses on UIV and UIV + 1 were seen in the T9 UIV model, and the tensile stresses on the PLC were increased in both UIV models. The surgical restoration of global sagittal spinal alignment may reduce the stresses on both the vertebrae of the UIV and UIV + 1. Posterior tethers successfully decreased both compressive stresses on the anterior column and tensile stresses on the PLC of adjacent segment in both UIV levels, though the amount of reduction in compressive stress on the anterior column was limited in T9 UIV model.

One of the limitations of the present study was that the simulation is performed with only one condition of bone strength and BMI.

However, in this study, we performed simulations using various conditions including 3 spine alignments and 2 UIV levels. The results consistently showed that the posterior tethers significantly decreased the tensile stress of ISL/SSL in both UIV models.

Further study on the optimal tightening strength of tethers in an osteoporotic bone model and obese model may necessary to improve this procedure for the prevention of the development of PJK.

## Conclusion

The compressive stresses of vertebral anterior column on the UIV and UIV + 1 vertebra in the UIV T9 model, and the tensile stresses on the PLC in both the T1 and T9 UIV models were significantly greater than in the no-fixation control. Reinforcement of the PLC by posterior tethers may reduce the development of PJK when spinal fusion is performed with a proximal thoracic UIV, while both posterior tether and vertebral augmentation may be necessary to reduce the development of PJK when the fusion has a lower thoracic UIV.

## Supplementary information

Supplementary Information.
