# Supplementary material for: The effect of posterior tethers on the biomechanics of proximal junctional kyphosis: The whole human finite element model analysis
Source: Sci Rep. 2020 Feb 26;10:3433. doi: 10.1038/s41598-020-59179-w (PMC7044281; doi:10.1038/s41598-020-59179-w)
Supplement: Supplementary file 1 — Supplementary Information. [file 41598_2020_59179_MOESM1_ESM.pdf]

The effect of posterior tethers on the biomechanics of proximal junctional kyphosis: The whole human finite element model analysis

Authors:

Mitsuru Yagi M.D., Ph.D.,[1,2], Yuko Nakahira,[3], Kota Watanabe M.D., Ph.D.,[1], Masaya Nakamura M.D., Ph.D.,[1], Morio Matsumoto M.D., Ph.D.,[1], Masami Iwamoto Dr. Eng.[3]

1. Department of Orthopedic Surgery, Keio University School of Medicine
2. Department of Orthopedic Surgery, National Hospital Organization Murayama Medical Center
3. Toyota Central R&D Labs Inc.

Affiliations:

Mitsuru Yagi and Yuko Nakahira contributed equally to this work

Corresponding author:

#1 Mitsuru Yagi M.D., Ph.D.,

35, Shinanomachi, Shinjyuku, Tokyo 160-8582, Japan

Tel: +81.3.3353.1211; fax: +81.3.3353.1211.; e-mail: yagiman@keio.jp

#2 Masami Iwamoto, Dr. Eng.

Koraku Mori Building 10F, 1-4-14, Koraku, Bunkyo-ku, Tokyo 112-0004, Japan

Tel: +81.561.71.8007; fax: +81.561.63.6042.; e-mail: iwamoto@mosk.tytlabs.co.jp

This study was approved by the appropriate institutional review board.

Supplemental Figure 1

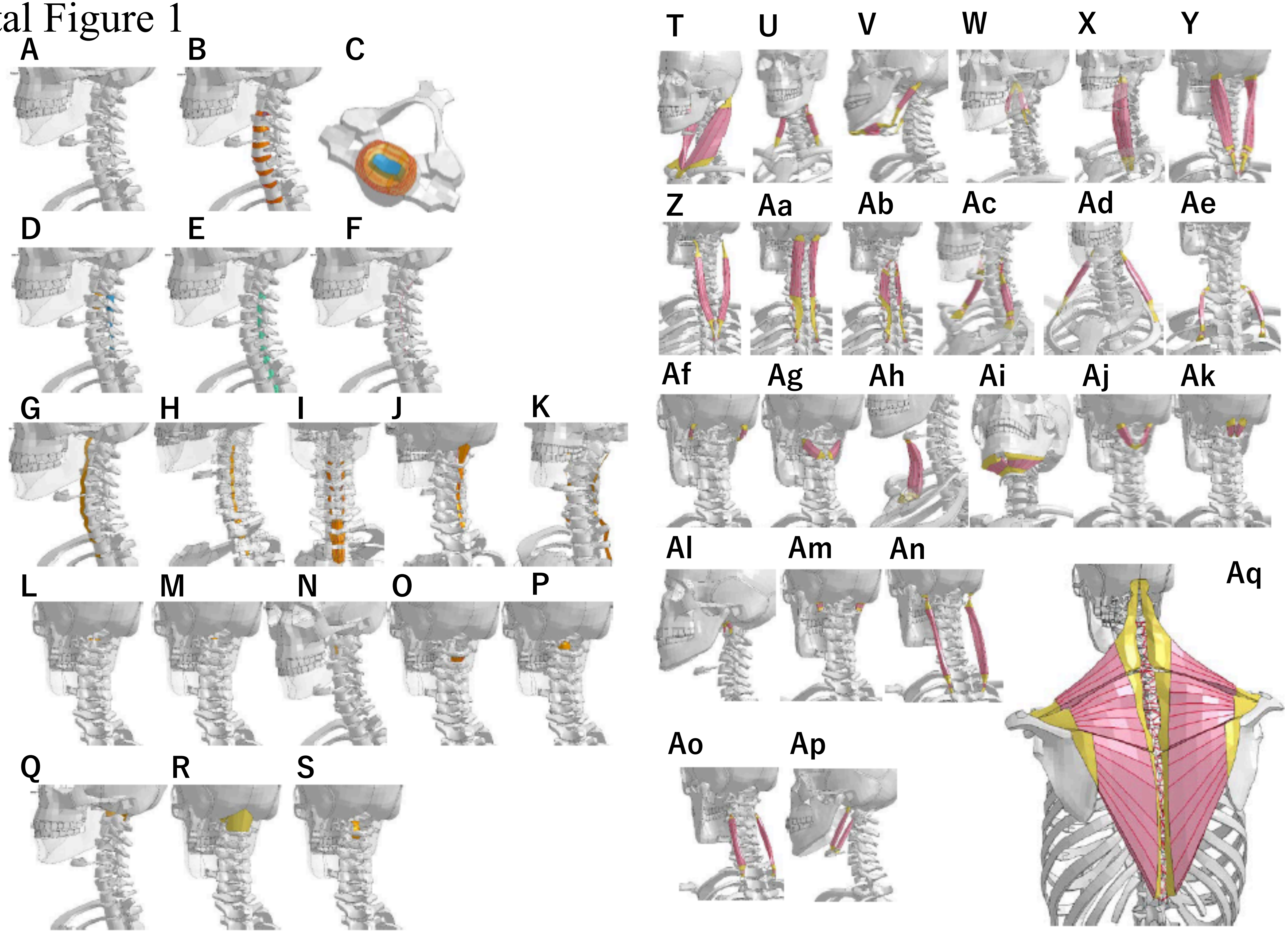

Supplemental Figure 2

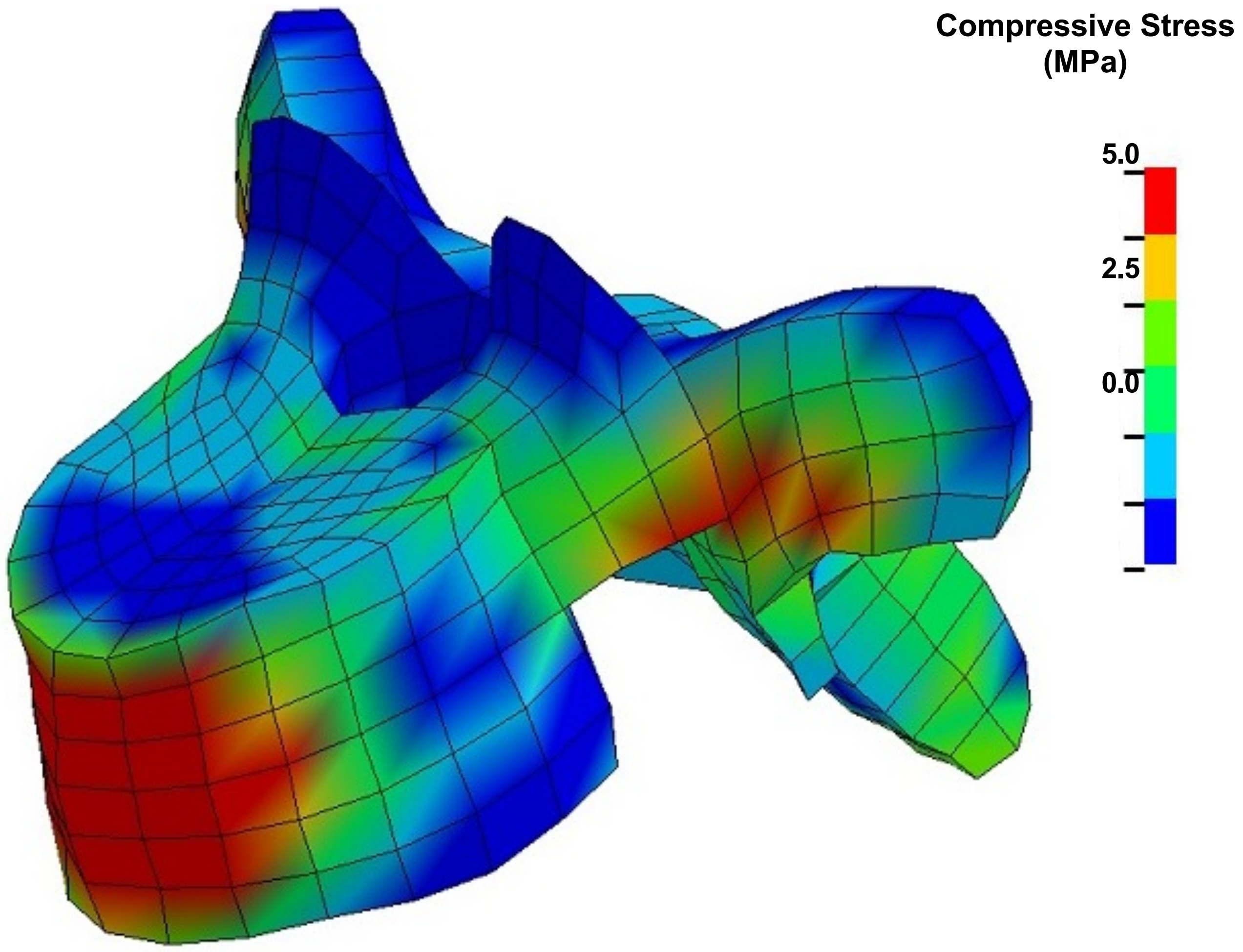

Supplemental Figure 3

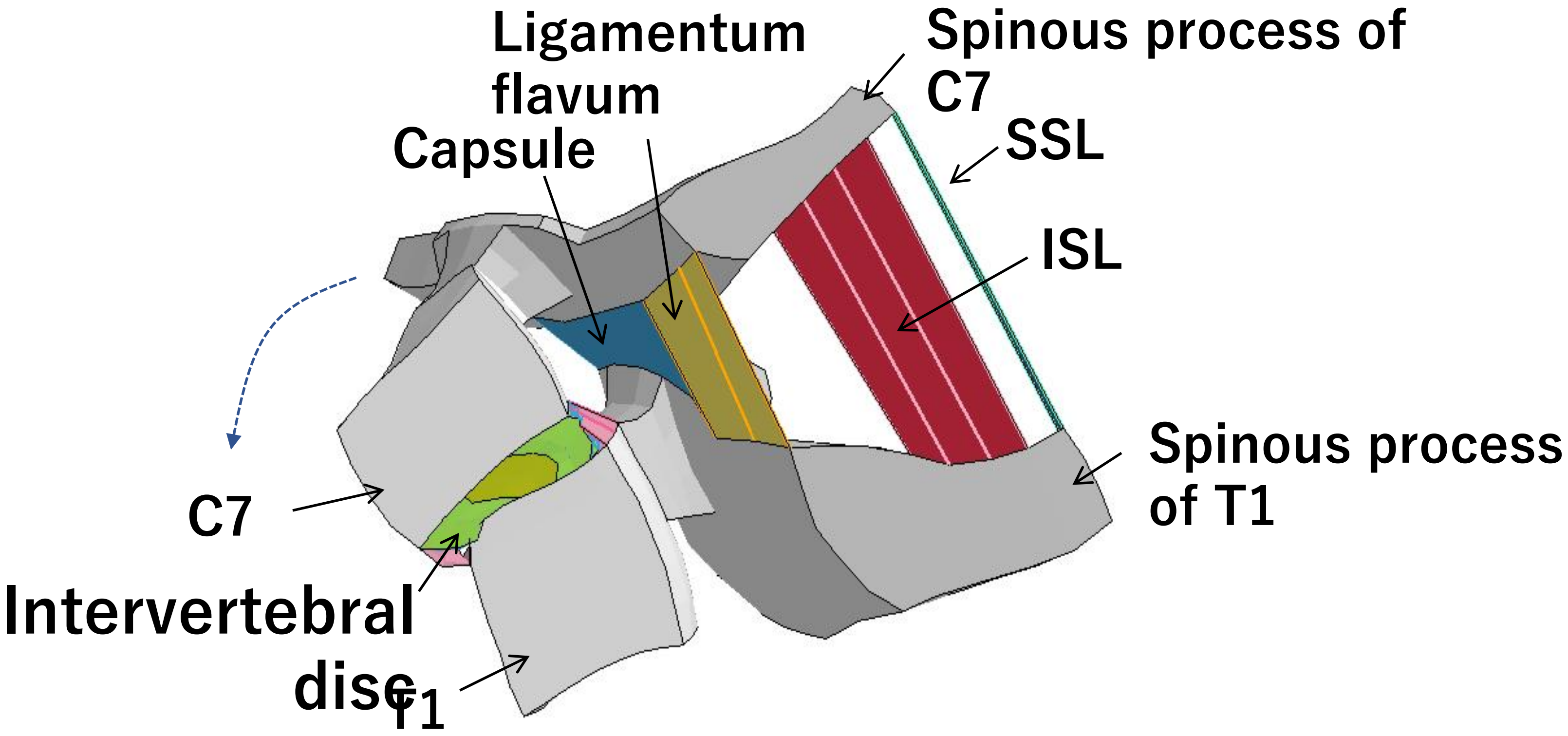

Supplemental Figure 4

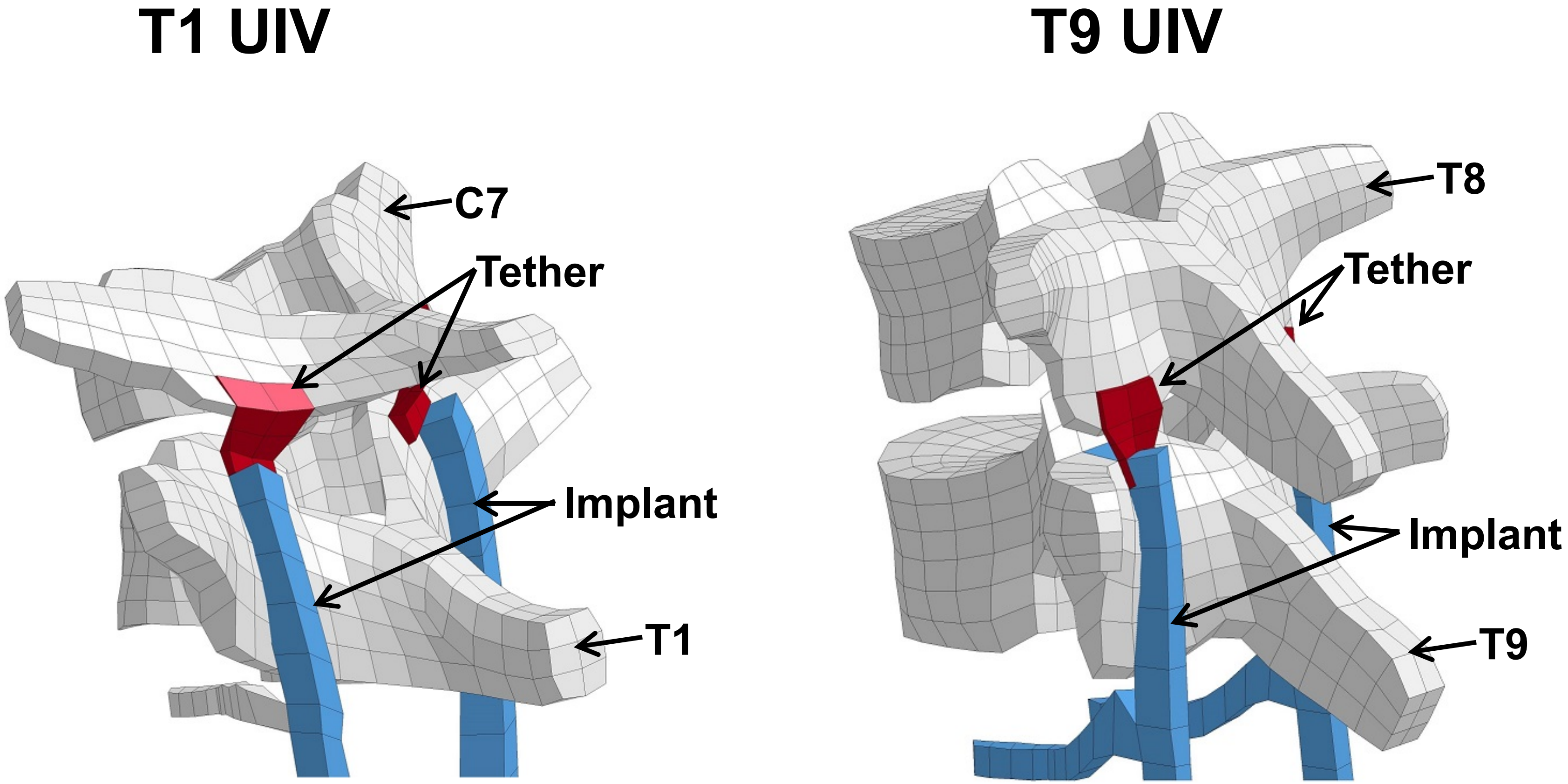

SUPPLEMENTAL FIGURE LEGENDS

Supplemental figure 1. Schematic view of the materials in a human body FE model used in this study.

A, Cervical vertebrae, rib cage, and hyoid; B, C, Intervertebral discs; D, Cartilage of facet joints; E, Spinal cord; F, Vertebral artery; G, Anterior cruciate ligament; H, Posterior cruciate ligament; I, Ligamentum Flavum; J, Ligamentum Nuchae; K, Inter-transverse ligament; L, Alar ligament; M, ligamentum apicis dentis; N, Anterior atlantoaxial ligament; O, Posterior atlantoaxial ligament; P, Cruciate ligament of atlas; Q, Anterior atlanto-occipital membrane; R, Posterior atlanto-occipital membrane; S, Tectorial membrane; T, Sternocleidomastoid muscle; U, Middle scalene muscle; V, Digastric muscle; W, Longus capitis muscle; X, Longus coli muscle; Y, Splenius capitis muscle; Z, Splenius cervicis muscle; Aa, Semispinalis capitis muscle; Ab, Semispinalis cervicis muscle; Ac, Anterior scalene muscle; Ad, Posterior scalene muscle; Ae, Iliocostalis cervicis muscle; Af, Superior obliquus capitis muscle; Ag, Inferior obliquus capitis muscle; Ah, Sternohyoid muscle; Ai, Mylohyoid muscle; Aj, Rectus capitis posterior major; Ak, Rectus capitis posterior minor; Al, Rectus capitis anterior; Am, Rectus capitis lateralis; An, Longissimus capitis muscle; Ao, Longissimus cervicis; Ap, Stylohyoid muscle; Aq, Trapezius muscle.

Supplemental figure 2. Compressive stress distributions of cortical part in the thoracic spinal column of the human body FE model

Compressive stress distributions of cortical part in the thoracic spinal column model. High compressive stresses were observed on the anterior surface of the vertebral body.

Supplemental figure 3. Deformation of ligaments and disc between C7 and T1 of the human body FE model

T1 spinal tilt 20 degrees, UIV at T1, and gravity load for 0.7 seconds under 20% muscle contracture. Only the right half is shown to reveal the deformation of the intervertebral disc. The broken line arrow in the figure is the movement of C7 with respect to T1. When bending forward, the anteversion angle of C7 becomes larger than T1, whereby the distance between the anterior column of C7 and T1 decreases, and at the same time, the distance between the spinous processes increases, stretching the supraspinous ligament (SSL) and intraspinous ligament (ISL).

Supplemental figure 4. Schematic view of T1 UIV and T9 UIV model used in this study

The material properties of the posterior elastic tether were obtained from the previous report [40] (Young’s module: 3.5 GPa, Poisson’ ratio: 0.4, thickness: 0.5 mm, width: 7 mm).
